# Diabète et infarctus: uniquement une histoire de cœur?

**DOI:** 10.11604/pamj.2015.22.239.7659

**Published:** 2015-11-13

**Authors:** Youssef Kort, Naziha Khammassi

**Affiliations:** 1 Service de Médecine Interne, Hôpital Razi, Faculté de médecine de Tunis, La Manouba 2010, Tunisie

**Keywords:** Diabète, infarctus osseux, complications ostéo-articulaires, Diabetes, bone infarction, osteo-articular complications

## Image en medicine

Les complications ostéo-articulaires du diabète de type 2 sont diverses parmi lesquelles certaines sont fréquentes comme la capsulite rétractile et la cheiro arthropathie diabétique. D'autres sont méconnues et difficilement rattachées au diabète. Nous rapportons l'observation d'une femme de 56 ans aux antécédents de diabète de type 2 insulino nécessitant depuis 6 ans mal équilibré (HbA1C à 10%) et qui se plaignait depuis 2 ans de douleurs de l'épaule et du bras gauches d'intensité moyenne à modérée. L'interrogatoire ne révélait pas de fièvre ou de signes d'altération de l'état général. A l'examen, il existait une douleur à la palpation du tiers supérieur du bras gauche sans arthrite de l'épaule ni limitation à la mobilisation active et passive de celle-ci. A la biologie, le bilan phosphocalcique, les phosphatases alcalines, le bilan rénal et la numération formule sanguine étaient sans anomalies. Il n'y avait pas de syndrome inflammatoire biologique. La radiographie de l'épaule était normale. A la radiographie du bras, il existait une image diaphysaire ostéocondensante irrégulière et en motte du tiers proximal de l'humérus sans effraction de la corticale. Cet aspect était typique d'un infarctus osseux ancien bien que la localisation à l'humérus ne soit pas fréquente. L'infarctus osseux peut être favorisé chez l'adulte par un traitement corticoïde ou la consommation d'alcool. Plus rarement, il peut être révélateur d'une maladie de gaucher de l'adulte mais il reste idiopathique dans environ 50% des cas.

**Figure 1:**
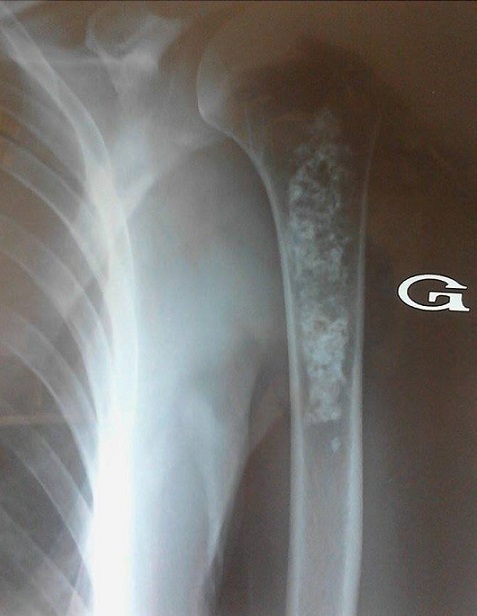
Radiographie du bras: image diaphysaire ostéocondensante irrégulière et en motte du tiers proximal de l'humérus sans effraction de la corticale

